# Magnetic resonance imaging features of myxoid leiomyoma of the vagina: A case report

**DOI:** 10.4103/0971-3026.54880

**Published:** 2009-08

**Authors:** Michele Scialpi, Giuseppe Benagiano, Sara Frati, Irene Piscioli, Francesco Barberini, Luciano Lupattelli

**Affiliations:** Department of Surgical, Radiological and Odontostomatological Sciences, Section of Diagnostic and Interventional Radiology, University of Perugia, S. Maria della Misericordia Hospital, S. Andrea delle Fratte, 06134 Perugia, Italy; 1 Department of Radiology, Budrio Hospital, AUSL Bologna, 40054 Budrio, Italy; 2 Department of Surgical, Radiological and Odontostomatological Sciences, Section of Oncologic Surgery, Perugia University, S. Maria della Misericordia Hospital, S. Andrea delle Fratte, 06134 Perugia, Italy

**Keywords:** Leiomyoma, magnetic resonance imaging, vagina

## Abstract

We report a rare case of a voluminous vaginal myxoid leiomyoma in a 27-year-old nulliparous woman. Magnetic Resonance Imaging (MRI) revealed a mass arising from the vagina, with inhomogeneous signal intensity on spin-echo T1W and T2W images. MRI accurately defined the tissue planes between the lesion and the adjacent structures and suggested its benign nature. The mass was completely resected by means of transvaginal approach and the diagnosis of myxoid leiomyoma was confirmed histologically. To the best of our knowledge, this is the first report describing the MRI features of vaginal myxoid leiomyoma.

## Introduction

Leiomyoma is the most common benign mesenchymal tumor of the vagina in adult women.[1] Since the first report by Denys de Leyden in 1773, approximately 300 cases have been reported to date.[2] Although vaginal leiomyoma is a rare neoplasm, the clinical features have been widely described.[3]

We report a rare case of a voluminous myxoid leiomyoma arising from the vagina. The emphasis is on the value of MRI in the diagnosis and management of such a lesion.

## Case Report

**Figure 1:**
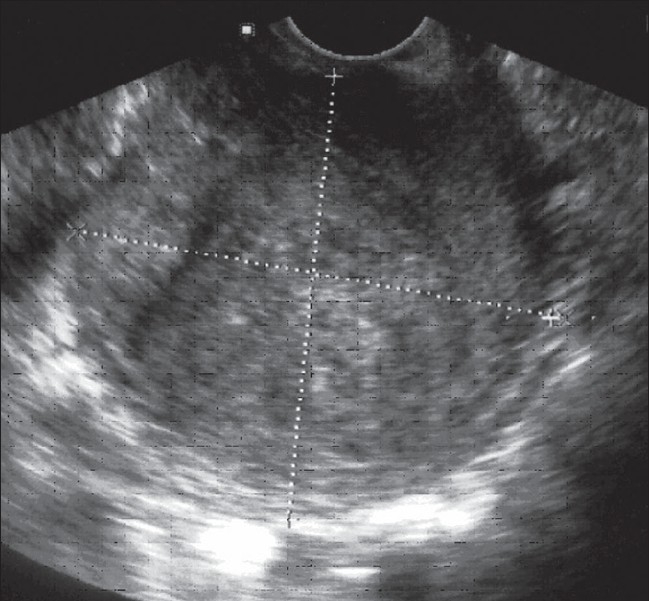
TVUS image shows a voluminous, round (approximately 7 cm in diameter), heterogeneous pelvic mass with well-defined contours arising from the anterior wall of the vagina

A 27-year-old nulliparous woman was admitted to our hospital for dyspareunia, pressure symptoms on the urinary tract, vaginal pain, and fetid vaginal discharge for 20 days. On vaginal exploration there was a palpable, soft mass in the anterior wall of the vagina. Transvaginal Ultrasound (TVUS) showed a round (approximately 7 cm in diameter) heterogeneous pelvic mass, with well-defined contours [Figure 1], arising from the anterior wall of the vagina. The mass displaced the urinary bladder anteriorly and superiorly.

In order to confirm the tumor origin and its relationships with adjacent organs, we performed MRI using a 1.5-T superconducting system (Signa Advantage; GE Medical Systems, Milwaukee, WI, USA). Sagittal, axial, and coronal T2W (TR / TE = 5000 / 100) fast spin-echo images and axial T1W (TR / TE = 460 / 14) spin-echo images, using a section of 7 mm and an intersection gap of 1 mm, were obtained.

**Figure 2 (A, B):**
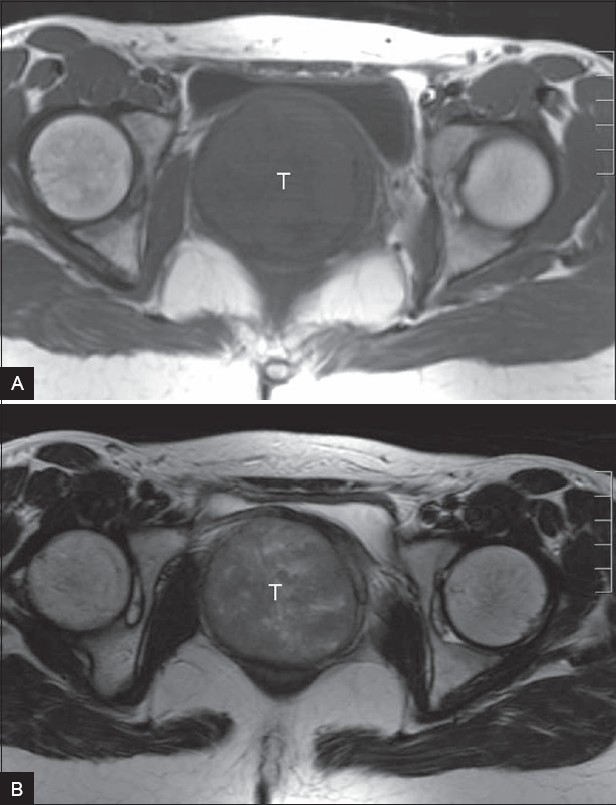
Axial T1W (TR / TE = 460 / 14) MRI (A) shows a wellcircumscribed, isointense, relatively heterogeneous, rounded (7.5 cm in diameter) pelvic mass (T) that displaces the urinary bladder anteriorly and compresses it. On the axial T2W (TR / TE = 5000 / 100) MRI (B), the mass (T) has an isointense signal with internal hyperintense irregular areas

**Figure 3:**
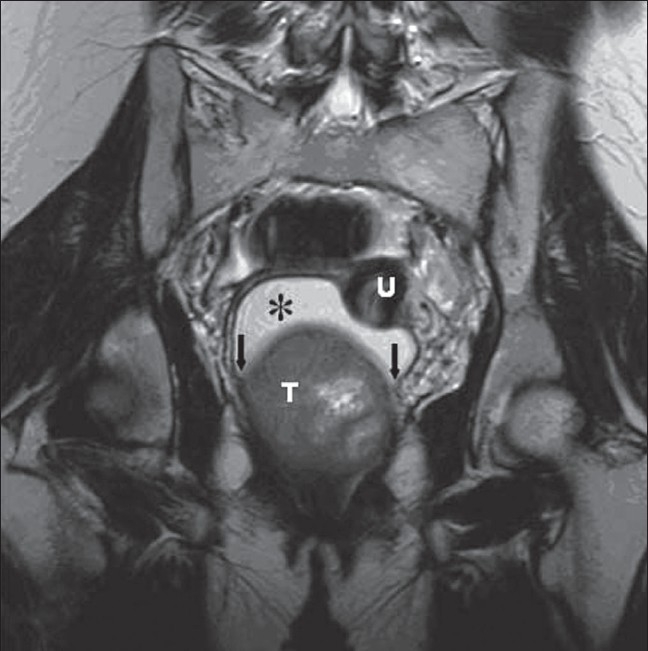
Coronal T2W (TR / TE = 5000 / 100) MRI shows the mass (T), displacing the uterus (U) cranially, and causing distension of the fornices and enlargement of the vaginal lumen* with high signal intensity. Note the acute angles (arrows) between the tumor and vaginal wall, suggesting its vaginal origin

Axial T1W MRI revealed a well-circumscribed, isointense, relatively heterogeneous, rounded (7.5 cm in diameter) pelvic mass that displaced and compressed the urinary bladder anteriorly [Figure 2a]. On axial T2W images, the mass showed isointense signal with internal hyperintense irregular areas [Figure 2b]. On coronal T2W images, the mass had high internal signal intensity; it caused displacement of the uterus superiorly and distension of the vaginal fornices and vagina [Figure 3]. The acute angles between the lesion and the vaginal wall suggested a vaginal origin of the lesion. No enlarged pelvic lymph nodes were revealed. A diagnosis of a benign solid tumor arising from the vagina was made.

**Figure 4:**
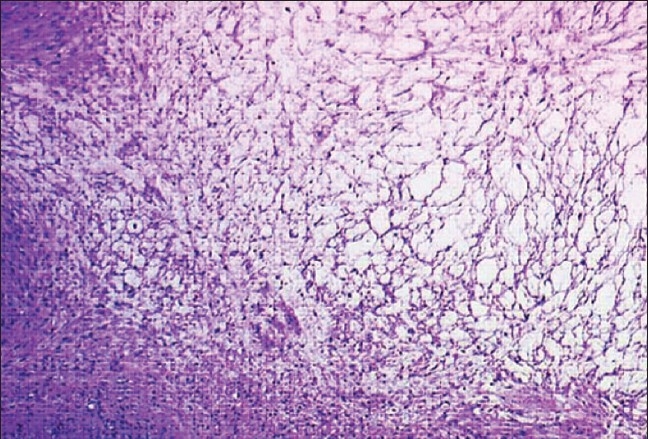
Photomicrograph reveals the typical proliferative pattern of myxoid leiomyoma. Abundant amorphous myxoid material is present between the smooth muscle cells (H&E; 40×)

Surgical excision by means of a transvaginal approach was performed. Gross examination revealed a well-circumscribed soft mass, with a maximum diameter of 7.5 cm. The histology showed the typical features of a myxoid leiomyoma [Figure 4]. Abundant amorphous myxoid material was present between the smooth muscle cells. The margins were circumscribed. Cytological atypia and mitotic figures were not found. Immunohistochemical studies showed the tumor cells to be positive for muscle-specific actin. The patient was discharged on the eleventh postoperative day. Six months later she was asymptomatic and voiding normally.

## Discussion

Vaginal leiomyomas may be up to 5 cm in size and may sometimes simulate malignancy (due to their size, unusual subtype, or the presence of degenerative changes). Knowledge of tumor features, especially with regard to tumor margins, relationship with adjacent structures, and size, is essential for differentiating between benign and malignant lesions preoperatively.

The value of MRI in the evaluation of vaginal masses has already been established.[4] On MRI, the appearance and signal characteristics of leiomyomas are varied[5] and depend on their histological patterns (i.e., the proportion of cellular fascicles, presence of degenerative changes, and the extracellular matrix).[6,7] Several histologic variants of uterine leiomyoma have been defined: cellular, mitotically active, hemorrhagic, epithelioid, atypical, and lipoleiomyoma.

MRI may be useful in the detection of ordinary leiomyoma. Only a few reports in the literature have described MRI findings in uterine myxoid leiomyoma. In a case reported by Cruz *et al*.,[8] MRI demonstrated the lesion as arising from the uterus. Contrast-enhanced Computed Tomography and MRI strongly suggested the cystic and myxoid content of the tumor. In a study of 18 uterine leiomyomas with interstitial edema and highly vascular loose connective tissue, Okizura *et al*.[9] observed an area of heterogeneous high signal intensity on T2W images that enhanced after gadolinium-DTPA administration. The women were premenopausal and none had received exogenous hormonal therapy before surgery. In all cases, areas of low to intermediate signal intensity on T2W images and enhancement after gadolinium-DTPA, corresponding to the final and early stages of myxoid leiomyoma, respectively, were revealed.

Usually, vaginal leiomyoma shows US and MRI features similar to those observed in the uterine counterpart.[10] However, the lesion may exhibit variable signal intensities on MRI, depending on its histopathologic changes.[11]

To the best of our knowledge, this is the first description of MRI findings in a myxoid leiomyoma of the vagina.

In the reported cases of vaginal leiomyomas [Table 1],[10–12] the diagnosis of ordinary leiomyoma was made by MRI and confirmed histologically. Only in a case described by Shimada *et al*.[11] did the MRI findings suggest a diagnosis of cellular leiomyoma, whereas histological examination revealed ordinary leiomyoma. The discrepancy between the MRI features and the histologic findings was related to the abundance of vessels in the vaginal leiomyoma, which caused hyperintensity on T2W images and marked contrast enhancement on the early dynamic MRI.[11]

**Table 1:** MRI of leiomyoma of the vagina: review of the literature

Case/Author	Age (years)	MRI findings	Histological type
Ruggieri *et al.*[10]	42	Mass arising from the anterior wall of the vagina, displacing the vaginal canal posteriorly, with a homogeneous signal similar to that of the uterine myometrium on T1W and T2W images.*	Ordinary L.
Shimada *et al.*[11]	37	Vaginal tumor, 2.2 cm in diameter, with a smooth contour and of homogeneous low signal intensity on the T1W images and homogeneous high signal intensity on T2W images. Dynamic contrast-enhanced MRI showed early homogenous marked contrast enhancement and delayed staining.	Ordinary L.
Shadbolt *et al.*[12]	30	Well-circumscribed, homogeneous, solid 4 × 4.5 × 5.6-cm mass in the anterior vaginal wall, separate from the cervix, urethra, and rectum, with relatively low signal intensity on T1W and T2W images.*	Ordinary L.
Shadbolt *et al.*[12]	40	Well-circumscribed, homogeneous mass of relatively low signal intensity on T1W and T2W images, arising from the right anterior vaginal wall. The mass demonstrated uniform contrast enhancement.	Ordinary L.

L: leiomyoma.

* Intravenous gadolinium-DTPA was not administered.

In our case, the T2W coronal images established the tumor origin by demonstrating acute angles between the lesion and the vagina. In spite of the large size and the inhomogeneous signal intensity of the mass on T1W and T2W images, MRI suggested the benign nature of the tumor on the basis of its regular contours and the absence of invasion of the adjacent pelvic organs. Also, MRI provided useful information for conservative surgical treatment (enucleation of the tumor).

## References

[1] Nucci MR, Fletcher CD (2000). Vulvovaginal soft tissue tumors: Update and review. Histopathology.

[2] Haberal A, Gunes M, Kayikcioglu F, Ozturkoglu E, Katas B, Demir OF (2005). Leiomyoma of the vagina: A case report. J Turkish German Gynecol Assoc.

[3] Woodruff JD, Julian CG, Puray T, Mermut S, Katayama P (1973). The contemporary challenge of carcinoma in situ of the vulva. Am J Obstet Gynecol.

[4] Elsayes KM, Narra VR, Dillman JR, Velcheti V, Hameed O, Tongdee R (2007). Vaginal masses: Magnetic resonance imaging features with pathologic correlation. Acta Radiol.

[5] Aggarwal BK, Panwar S, Rajan S, Aggarwal A, Ahlawat K (2005). Varied appearances and signal characteristics of leiomyomas on MR imaging. Indian J Radiol Imaging.

[6] Hricak H, Tscholakoff D, Heinrichs L, Fisher MR, Dooms GC, Reinhold C (1986). Uterine leiomyoma: Correlation of MR, histopathologic findings and symptoms. Radiology.

[7] Hamlin DJ, Pettersson H, Fitzsimmons J, Morgan LS (1985). MR imaging of uterine leiomyoma and their complications. J Comput Assist Tomogr.

[8] Cruz M, Murakami T, Tsuda K, Kurachi H, Enomoto T, Kim T (2001). Myxoid leiomyoma of the uterus: CT and MRI features. Abdom Imaging.

[9] Okizuka H, Sugimura K, Takemori M, Obayashi C, Kitao M, Ishida T (1993). MR detection of degenerating uterine leiomyoma. J Comput Assist Tomogr.

[10] Ruggieri AM, Brody JM, Curhan RP (1996). Vaginal leiomyoma: A case report with MR findings. J Reprod Med.

[11] Shimada K, Ohashi I, Shibuya H, Tanabe F, Akashi T (2002). MR imaging of atypical vaginal leiomyoma. Am J Roentg.

[12] Shadbolt CL, Coakley FV, Qayyum A, Donat SM (2001). MRI of vaginal leiomyoma. J Comput Assist Tomogr.

